# Role of the EHD2 Unstructured Loop in Dimerization, Protein Binding and Subcellular Localization

**DOI:** 10.1371/journal.pone.0123710

**Published:** 2015-04-15

**Authors:** Kriti Bahl, Naava Naslavsky, Steve Caplan

**Affiliations:** Department of Biochemistry and Molecular Biology, the Fred and Pamela Buffett Cancer Center, The University of Nebraska Medical Center, Omaha, Nebraska, United States of America; Purdue University, UNITED STATES

## Abstract

The C-terminal Eps 15 Homology Domain proteins (EHD1-4) play important roles in regulating endocytic trafficking. EHD2 is the only family member whose crystal structure has been solved, and it contains an unstructured loop consisting of two proline-phenylalanine (PF) motifs: **KPF**RKL**NPF**. In contrast, despite EHD2 having nearly 70% amino acid identity with its paralogs, EHD1, EHD3 and EHD4, the latter proteins contain a single KPF or RPF motif, but no NPF motif. In this study, we sought to define the precise role of each PF motif in EHD2’s homo-dimerization, binding with the protein partners, and subcellular localization. To test the role of the NPF motif, we generated an EHD2 NPF-to-NAF mutant to mimic the homologous sequences of EHD1 and EHD3. We demonstrated that this mutant lost both its ability to dimerize and bind to Syndapin2. However, it continued to localize primarily to the cytosolic face of the plasma membrane. On the other hand, EHD2 NPF-to-APA mutants displayed normal dimerization and Syndapin2 binding, but exhibited markedly increased nuclear localization and reduced association with the plasma membrane. We then hypothesized that the single PF motif of EHD1 (that aligns with the KPF of EHD2) might be responsible for both binding and localization functions of EHD1. Indeed, the EHD1 RPF motif was required for dimerization, interaction with MICAL-L1 and Syndapin2, as well as localization to tubular recycling endosomes. Moreover, recycling assays demonstrated that EHD1 RPF-to-APA was incapable of supporting normal receptor recycling. Overall, our data suggest that the EHD2 NPF phenylalanine residue is crucial for EHD2 localization to the plasma membrane, whereas the proline residue is essential for EHD2 dimerization and binding. These studies support the recently proposed model in which the EHD2 N-terminal region may regulate the availability of the unstructured loop for interactions with neighboring EHD2 dimers, thus promoting oligomerization.

## Introduction

The C-terminal Eps15 homology domain-containing (EHD) proteins are involved in a variety of endocytic membrane regulatory events [1]. All four EHDs share a characteristic domain architecture that includes a C-terminal Eps15 Homology (EH) domain with a positively charged electrostatic surface that selectively binds to proteins containing an asparagine-proline-phenylalanine (NPF) motif followed by acidic residues [2–5]. In addition, each EHD protein contains a dynamin-like G-domain that binds ATP and catalyzes its hydrolysis [6–9].

The most diverse EHD both in its sequence homology and function is EHD2 [10]. EHD2 has been reported to regulate a variety of important functions that include sarcolemmal repair [11], myoblast fusion [12,13], and control of Rac1 and the actin cytoskeleton [14–17]. In contrast to EHD1, EHD3 and EHD4, all of which play roles in regulating endocytic transport from sorting and recycling endosomes [1,18,19], EHD2 is recruited to the plasma membrane by phosphatidylinositol (4,5)-bisphosphate (PI(4,5)P2) [20] where it interacts with caveolin and regulates caveolar mobility [14,21,22].

**Fig 1:**
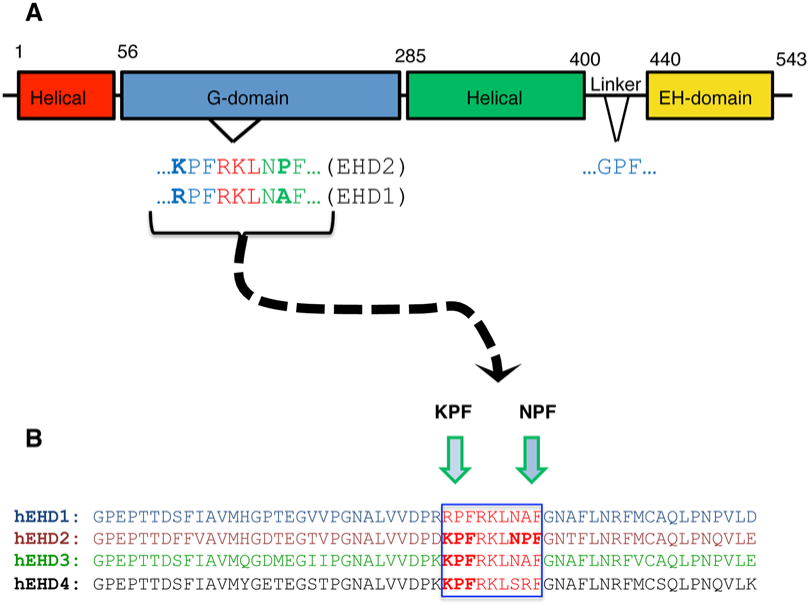
EHD protein domain architecture and sequence homology. (A) The Eps15 Homology Domain (EHD) proteins have a conserved domain architecture comprised of four domains: two helical domains, a G-domain, and a C-terminal EH domain. The G-domain of EHD2 contains an unstructured loop containing the following amino acid sequence, KPFRKLNPF, which is required for oligomerization. (B) The four EHD isoforms share 67–86% residue identity. The amino acid sequence alignment of the unstructured loop for all four EHDs (see green frame) shows that only EHD2 has two successive **PF** motifs: N**PF** and K**PF**, whereas the other EHD proteins have only one **PF** motif.

The mouse EHD2 crystal structure indicates that this protein contains a partially conserved region with two proline-phenylalanine motifs KPFRKLNPF in an unstructured flexible loop near the G-domain [9] (Fig 1A). This unstructured KPFRKLNPF region was proposed to link EHD2 dimer pairs through interactions with neighboring EH domains [9]. Recent studies provide support for the notion that both PF motifs may be important for EHD2 localization and function [22], in addition to the N-terminal region of EHD2 [23]. However, the degree to which each of these two closely situated PF motifs impacts EHD2 function, and particularly how each motif affects dimerization and interactions with binding partners remains unknown.

The function of the PF motifs in the unstructured loop in EHD proteins is further complicated because while EHD2 has dual PF motifs in its KPFRKLNPF sequence, the other three EHD paralogs all contain only a single PF motif (Fig 1B); either a KPF or RPF motif at the N-terminal side of the unstructured loop. Indeed, both EHD1 and EHD3 have a NAF motif (instead of NPF), whereas EHD4 has a SRF motif.

In this study, we set out to determine the significance of EHD2’s KPF and NPF motifs for its ability to dimerize, interact with protein partners and localize to caveolae. We demonstrate here that when the EHD2 NPF is mutated to NAF, there is loss of dimerization and protein binding, but this motif has little impact on EHD2 localization in the cell. On the other hand, mutation of the NPF motif to APA has little or no impact on dimerization or partner binding, but shunts EHD2 localization from mostly at the plasma membrane to the nucleus. Examining EHD1 as a representative of the other three more closely related EHDs, we found that the single RPF motif of EHD1 is required for (i) its dimerization, (ii) interaction with protein partners, and (iii) its localization to tubular recycling endosomes. Overall, these data indicate that the proline and phenylalanine residues that comprise EHD2’s NPF motif regulate different functions, suggesting that this motif may have additional roles to its proposed interactions with neighboring EHD2 EH domains.

## Materials and Methods

### Cells, Reagents and Antibodies

HeLa cells (cervical cancer cell line, ATCC-CCL2; Manassas, VA) were maintained in high glucose Dulbecco’s Modified Eagle’s Medium supplemented with 10% (v/v) fetal bovine serum (Sigma Aldrich), 200 U/ml penicillin, 200 μg/ml streptomycin, 2 mM L-glutamine, and 10 mM HEPES. All media reagents, except for the fetal bovine serum, were from Invitrogen. The following antibodies were used in this study: rabbit anti-caveolin1 (Cell Signaling Technology), Alexa-Fluor-568-conjugated goat anti-rabbit IgG (H+L) (Molecular Probes).

### Recombinant DNA Constructs

Cloning of EHD1, EHD2, EHD3, EHD4, MICAL-L1, Syndapin2 into yeast two-hybrid vectors pGBKT7 and pGADT7, as well as cloning of GFP-Myc–EHD1, its siRNA resistant version, and GFP-Myc-EHD2 is described in (Caplan et al., 2002; Sharma et al., 2009; Giridharan et al.,2013). Two-hybrid control vectors (Gal4ad-SV40 large T-antigen and Gal4bd-p53) were purchased from Clontech (Palo Alto). The following constructs were generated using a QuikChange Site-Directed Mutagenesis Kit (Stratagene): EHD2 (NPF-to-NAF), EHD2 (NPF-to-NPA), EHD2 (NPF-to-APA), EHD2 (KPF-to-KAF), EHD2 (KPF-to-APA), and EHD1 (RPF-to-APA) in yeast-two hybrid vectors and in the GFP-Myc-EHD1 and GFP-Myc-EHD2 vectors (mammalian expression vectors). The following mutations were also introduced by site-directed mutagenesis: GFP-Myc-EHD2 (NPF-to-NPY) and GFP-Myc-EHD2 (NPF-to-NFP).

### Yeast Two Hybrid

The yeast two-hybrid assay was done as described previously [24]. Briefly, the *Saccharomyces cerevisiae* strain, AH109 (BD Biosciences), was maintained on YPD agar plates. A loop full of yeast was grown overnight at 30°C and shaking at 250 rpm in liquid YPD media. The transformation was done by the lithium acetate procedure as described in the instructions for the MATCHMAKER two-hybrid kit (BD Biosciences). For colony growth assays, AH109 co-transformants were streaked on plates lacking leucine and tryptophan. Cells were left to grow at 30°C, usually for three days, or until the colonies were large enough for further assays. An average of three to four colonies were selected and suspended in water, equilibrated to the same optical density as other colonies from different transformants at 600 nm, and replated on plates lacking leucine and tryptophan (+HIS, -2 plates), as well as plates lacking histidine (-HIS, -3 plates). The positive control for interactions was co-transformed p53 and SV40.

### Confocal microscopy imaging

HeLa cells were grown on cover slips and fixed with 4% (v/v) paraformaldehyde in PBS. The fixed cells were then incubated with primary antibodies prepared in staining solution [0.2% (w/v) saponin and 0.5% (w/v) bovine serum albumin (BSA) in PBS] for 1 h at room temperature. After washing in PBS, the cells were incubated with the appropriate fluorochrome-conjugated secondary antibody mixture in staining solution for 30 minutes at room temperature. Images were acquired with a Zeiss LSM 5 Pascal confocal microscope (Carl Zeiss, Thornwood, NY) using a 63 X 1.4 numerical aperture objective with appropriate filters.

### Structured illumination microscopy (SIM) imaging

SIM images were collected on samples obtained as described above with a Zeiss ELYRA PS.1 illumination system (Carl Zeiss Microimaging) using a 63 X objective lens with a numerical aperture of 1.4 at room temperature as we have previously done [25,26]. Three orientation angles of the excitation grid were acquired for each z plane, with z spacing of 110 nm between planes. SIM processing was performed with SIM module of the ZEN BLACK software (Carl Zeiss Microimaging).

### Transfections

HeLa cells, plated on 6 well-plates, were transfected using 6 μl X-tremeGENE 9 (Roche Applied Sciences) or 6 μl Lipofectamine 2000 (Invitrogen) with 2 μg of DNA according to manufacturer’s protocol.

siRNA duplexes for EHD1 (base pairs gaaagagatgcccaatgtc, Dharmacon) were transfected using Oligofectamine (LifeTechnologies) to perform knockdown studies as previously described (Naslavsky et al., 2006).

### Transferrin uptake assays

HeLa cells grown on glass clover slips, were starved in DMEM media containing 0.5% bovine serum albumin (BSA) for 1 h, then “pulsed” with 1 μg/ml of Alexa Fluor 568–labeled transferrin (Tf-568) for 15 minutes and chased for 15 minutes in complete media, to allow Tf-568 recycle to the PM and be released. Cells were then fixed and imaged by confocal microscopy.

### Statistical analysis

Where appropriate, the data presented were analyzed by one-way ANOVA, with one star for *p* < 0.01, and three stars for *p* < 0.05.

## Results and Discussion

**Fig 2:**
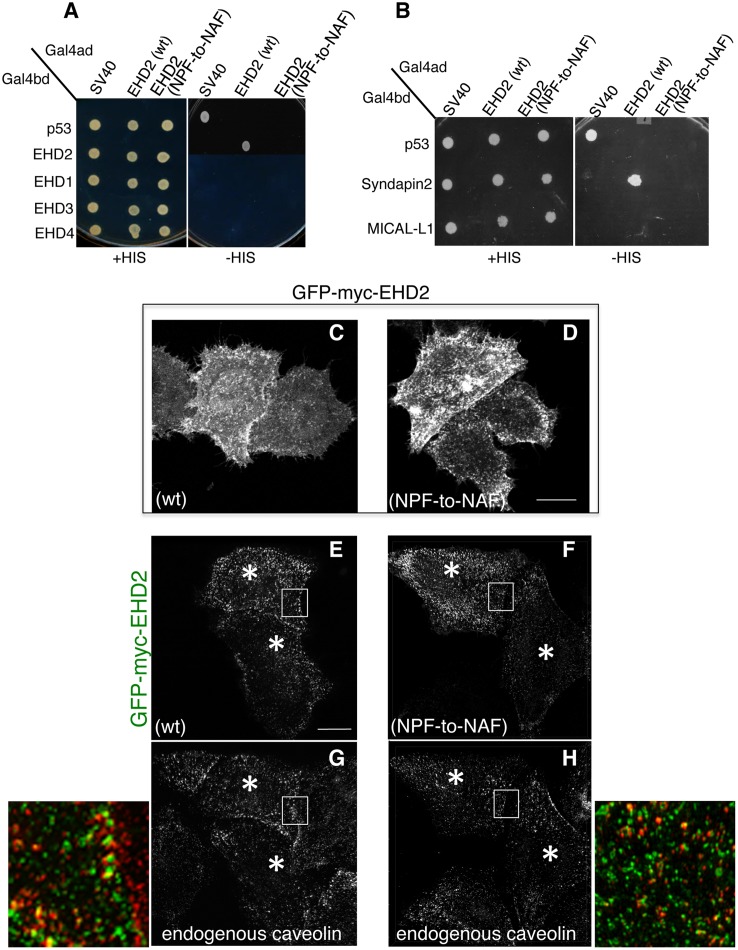
Modification of the EHD2 NPF motif to NAF impairs dimerization and binding with interaction partners, but does not affect EHD2 localization. (A) *S*. *cerevisae* yeast were co-transformed with the following Gal4 binding domain (Gal4bd) fusion constructs (see Materials and Methods):-p53 (control), -Syndapin-2 and -MICAL-L1. Gal4 activating domain (Gal4ad) fusion constructs were either-SV40 (control), -EHD2 (wt) or -EHD2 (NPF-to-NAF). Co-transformants were plated on non-selective (+HIS) and selective (-HIS) agar plates. (B) As in (A), co-transformation was with Gal4bd-p53 (control), -EHD2 (wt), -EHD1 (wt), -EHD3 (wt), and -EHD4 (wt) and with Gal4ad-SV40 (control), -EHD2 (wt) and -EHD2 (NPF-to-NAF). (C–H) HeLa cells were grown on coverslips, transfected with GFP-myc-EHD2 (wt) (C,E,G) or with GFP-myc-EHD2 (NAF-to-NPF) (D,F,H) and fixed. (C–D) are transfected cells imaged by confocal microscopy, whereas (E–H) are micrographs obtained by Structured Illumination Microscopy (SIM). Asterisks indicate transfected cells. Bar; 10 μm.

Unlike the other three EHDs, EHD2 homo-dimerizes but does not hetero-dimerize [20], and its homo-dimerization may be required for interaction with NPF-containing protein binding partners. Accordingly, our goal was to first determine the requirement of the EHD2 NPF motif for homo-dimerization (Fig 1A and 1B). By selective two-hybrid binding assay, we demonstrated that while wild-type EHD2 proteins homo-dimerize, they were incapable of interacting with wild-type EHD1, EHD3 or EHD4 (Fig 2A). On the other hand, when the EHD2 NPF motif was altered to NAF, the homo-dimerization was no longer observed.

EHD2 binds to select NPF-containing protein partners through its EH domain. As shown, wild-type EHD2 interacted with Syndapin2, but not MICAL-L1, even though both proteins contain NPF motifs followed by acidic clusters (Fig 2B). Moreover, this interaction could also be observed by co-immunoprecipitation (S2 Fig and S1 Table). However, the EHD2 NPF-to-NAF mutant lost its ability to interact with Syndapin2, suggesting that the EHD2 NPF motif is required for both homo-dimerization and for protein partner binding. Despite this data in yeast two-hybrid binding studies, the NAF mutant retained its ability to bind to Syndapin2 by co-immunoprecipitation, suggesting the indirect involvement of a bridging protein in cells (S2 Fig and S1 Table).

In our previous studies we have demonstrated that EHD2 is capable of interacting with PI(4,5)P2 at the plasma membrane without the need for interaction with other binding partners through its EH domain [20]. Moren et al. showed that EHD2 caveolar localization was independent of its protein-binding EH domain [22]. Indeed, we have demonstrated that the EH domain is not required for EHD2 (or EHD1) homo-dimerization (S1A–S1C Fig). Accordingly, we asked whether the EHD2 NPF-to-NAF mutant might still localize to the plasma membrane. As demonstrated in Fig 2C–2D, both wild-type and EHD2 NPF-to-NAF displayed primarily plasma membrane localization as demonstrated by confocal microscopy. Both the wild-type and EHD2 NPF-to-NAF mutant also partially co-localized to structures positive for caveolin-1, as observed by Structured Illumination Microscopy (Fig 2E–2H; see insets, asterisks indicate transfected cells). These data support the idea that the EHD2 NPF motif has an important role in EHD2 dimerization and protein binding, but that homodimerization is not required for normal localization to the plasma membrane and colocalization with caveolae.

**Fig 3:**
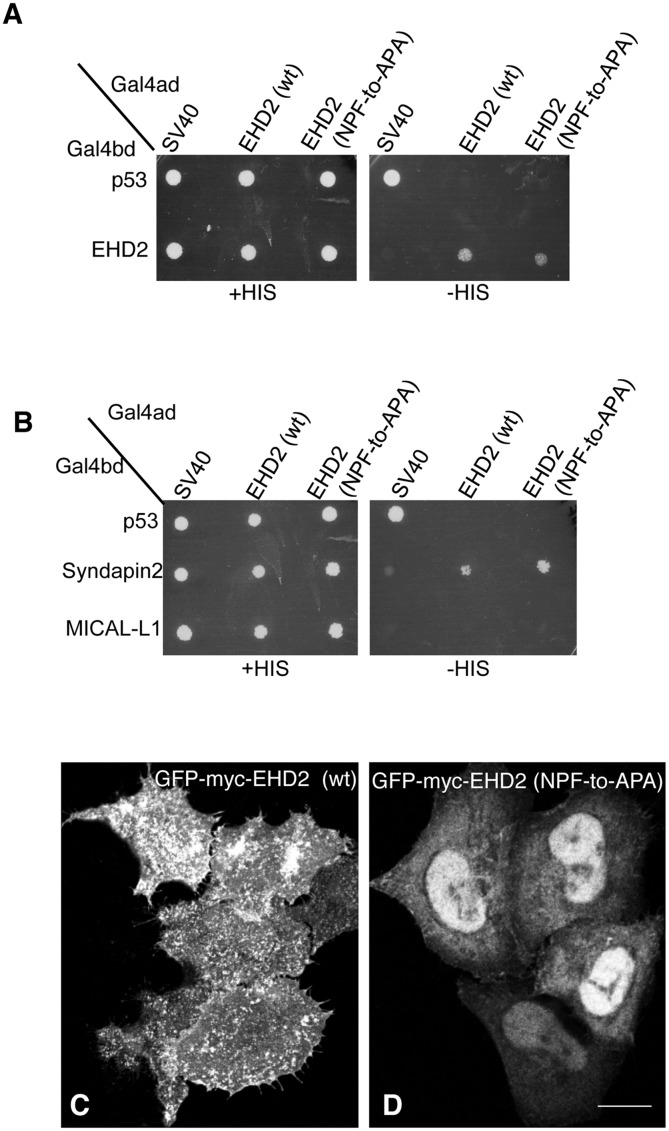
Modification of the EHD2 NPF motif to APA induces loss of plasma membrane localization, but does not affect interactions with binding partners. (A) *S*. *cerevisae* yeast were co-transformed with the following Gal4bd fusion constructs: Gal4bd-p53 (control), -Syndapin-2 and -MICAL-L1 along with Gal4ad fusion constructs Gal4ad-SV40 (control), -EHD2 (wt.) and -EHD2 (NPF-to-APA). (B) As in (A) co-transformation was with Gal4bd fusion constructs: Gal4bd-p53 (control), -EHD2 (wt) along with Gal4ad fusion constructs: Gal4ad-SV40 (control), -EHD2 (wt) and -EHD2 (NPF-to-APA). Co-transformants from A-B were plated on non-selective (+HIS) and selective (-HIS) agar plates. (C–D) HeLa cells were grown on coverslips, transfected with GFP-myc-EHD2 (C) or GFP-myc-EHD2 NPF-to-APA (D). Bar; 10 μm.

In a recent study by Moren et al., phenylalanine 128 of the EHD2 NPF motif (see Fig 1A) was replaced with alanine (NPF-to-NPA) and similarly, little change in EHD2 localization was reported [22]. Since we chose to initially disrupt the NPF motif by substituting the proline for alanine (NPF-to-NAF) to mimic the homologous sequence found in both EHD1 and EHD3, we decided to address the function of EHD2 when the proline residue of its NPF motif is left intact. To this aim we assessed homo-dimerization of EHD2 NPF-to-APA mutants as well as its interaction with Syndapin2 (Fig 3A and 3B). Surprisingly, unlike the EHD2 NPF-to-NAF mutant, the APA mutants retained both homo-dimerization and binding to Syndapin2. On the other hand, despite normal dimerization and binding, EHD2 NPF-to-APA displayed a dramatic relocation to the nucleus (Fig 3D). Although EHD2 has a bipartite nuclear localization sequence and many cells display a portion of their EHD2 in the nucleus (Fig 3C) [27], the NPF-to-APA mutant displayed a primarily nuclear localization (Fig 3D). Accordingly, despite the yeast two-hybrid binding, we were unable to detect EHD2 NPF-to-APA interactions with Syndapin2 by co-immunoprecipitation, likely due to the mutant’s mislocalization exclusively to the nucleus (S2 Fig and S1 Table).

**Fig 4:**
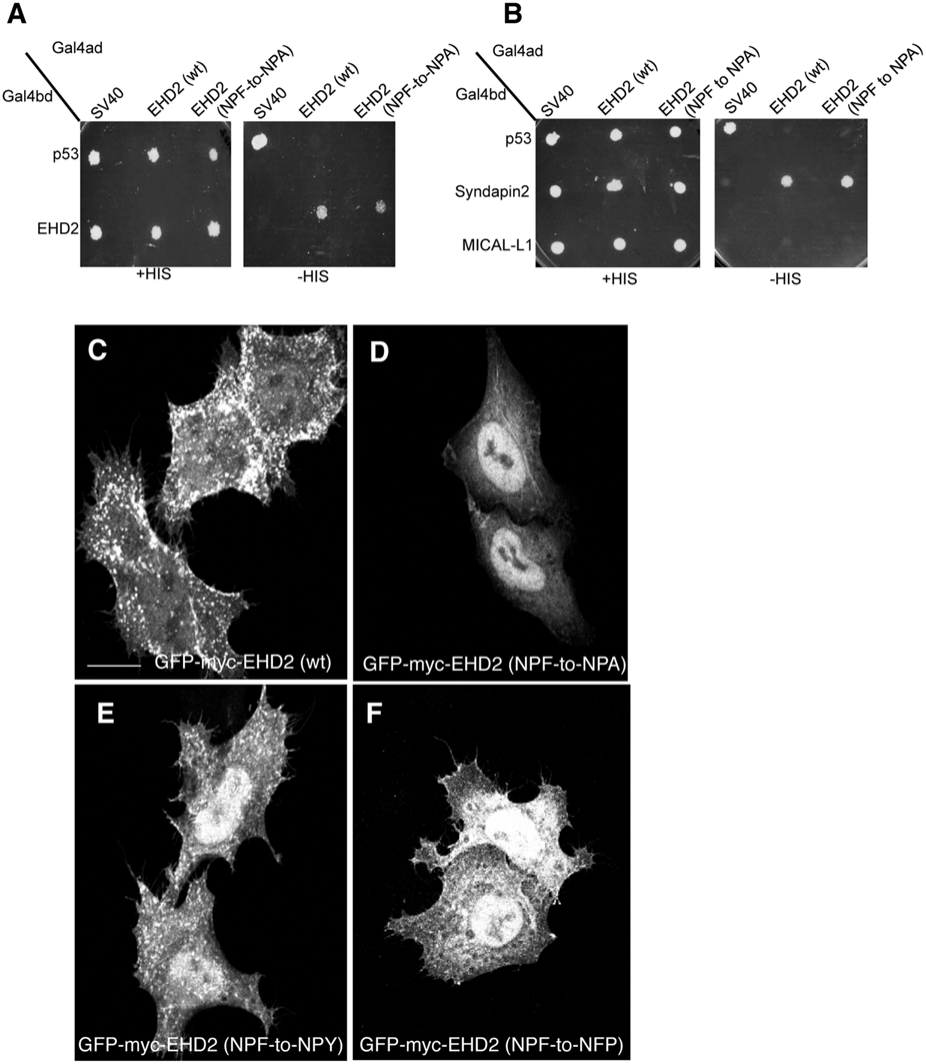
The NPF phenylalanine residue is responsible for the plasma membrane localization of EHD2. (A) *S*. *cerevisae* yeast were co-transformed with the following Gal4bd fusion constructs: Gal4bd-p53 (control), -Syndapin-2 and -MICAL-L1 along with Gal4ad fusion constructs: Gal4ad-SV40 (control), -EHD2 (wt) and -EHD2 (NPF-to-NPA). (B) As in (A), yeast were co-transformed with the Gal4bd fusion constructs: Gal4bd-p53 (control), -EHD2 (wt) along with Gal4ad fusion constructs: Gal4ad-SV40 (control), -EHD2 (wt.) and -EHD2 (NPF-to-NPA). Co-transformants were plated on non-selective (+HIS) and selective (-HIS) agar plates. (C–I) HeLa cells were grown on coverslips and transfected with: (C) GFP-myc-EHD2 (wt), (D) GFP-myc-EHD2 (NPF-to-NPA), (E) GFP-myc-EHD2 (NPF-to-NPY), (F) GFP-myc-EHD2 (NPF-to-NPA), (G) GFP-myc-EHD2 (NPF-to-NFP). Bar; 10 μm.

Since the Lundmark group used the more subtle EHD2 NPF-to-NPA mutant and did not report such relocation to the nucleus [22], we resolved to compare the EHD2 NPF-to-APA mutant to EHD2 NPF-to-NPA. As demonstrated in Fig 4A and 4B, similar to the APA mutant, EHD2 NPF-to-NPA continued to homo-dimerize and interact with Syndapin2. In addition, the subcellular distribution of EHD2 NPF-to-NPA was similar to that of EHD2 NPF-to-APA, with much of the mutant localizing to the nucleus and less observed at the plasma membrane (compare Fig 4C and 4D). These results suggested that the NPF phenylalanine residue either helps maintain EHD2 at the plasma membrane or possibly prevents it from transport to or export out of the nucleus. Although we cannot entirely reconcile the differences we observed in EHD2 NPF-to-NPA localization (largely nuclear) compared to those of Moren et al. (Fig 4B; GFP-EHD2 F128A) [22], not all the EHD2 relocalized to the nucleus, and the remaining EHD2 at the plasma membrane appeared to remain localized to caveolae (data not shown).

To specifically assess the role of phenylalanine 128, we engineered an EHD2 NPF-to-NPY mutant. Our rationale was that tyrosine is the closest residue to phenylalanine, with the only difference being the additional hydroxyl group on the aromatic ring. As shown, EHD2 NPF-to-NPY displayed an intermediate cellular distribution (compare Fig 4E with 4C and 4D). Although plasma membrane association was not abolished, a greater component of the EHD2 NPF-to-NPY EHD2 mutant was observed in the nucleus. However, the NPF-to-NPY mutant continued to homo-dimerize with wild-type EHD2 and Syndapin2 (S3 Fig and S1 Table). We then asked whether the spatial localization of phenylalanine 128 is essential for its function in facilitating EHD2 localization primarily to the plasma membrane. To address this, we engineered EHD2 NPF-to-NFP, flipping the proline and phenylalanine residues at positions 127 and 128. The subcellular localization of this NFP mutant was mostly in the nucleus (Fig 4F), although some EHD2 was clearly localized to the plasma membrane. In this case, the NPF-to-NFP mutant failed to either homo-dimerize or interact with Syndapin2 (S3 Fig and S1 Table). These data support the notion that phenylalanine 128 is essential for plasma membrane localization, and that its position within the unstructured loop is highly significant.

**Fig 5:**
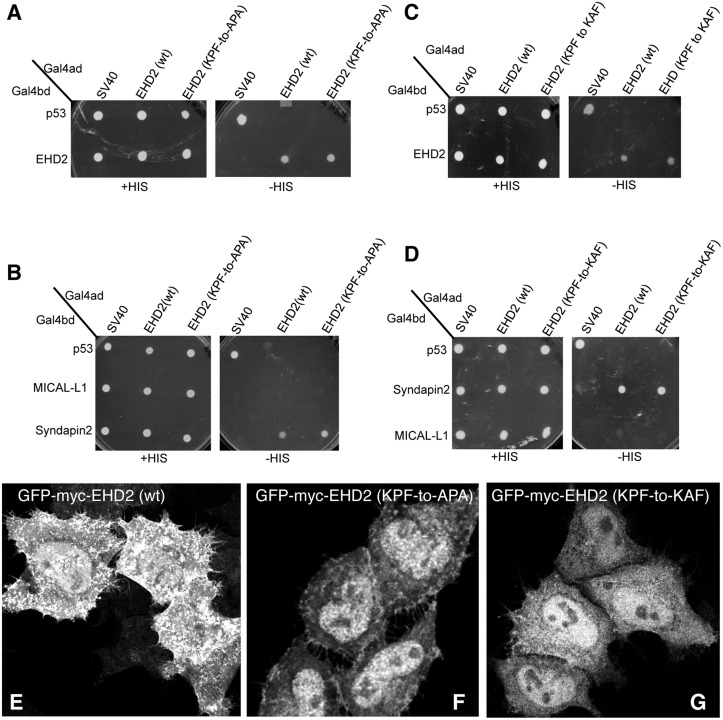
Disruption of the EHD2 KPF motif induces relocalization of EHD2 to the nucleus, but does not alter its binding ability. (A–D) *S*. *cerevisae* yeast were co-transformed with the following Gal4bd fusion constructs: Gal4bd-p53 (control), -Syndapin-2 and -MICAL-L1 along with Gal4ad fusion constructs: Gal4ad-SV40 (control), -EHD2 (wt), -EHD2 (KPF-to-APA) and—EHD2 (KPF-to-KAF). Co-transformants were plated on non-selective (+HIS) and selective (-HIS) media. (E–H) HeLa cells were grown on coverslips, transfected with GFP-myc-EHD2 (wt) (E), GFP-myc-EHD2 (KPF-to-APA) (F), or GFP-myc-EHD2 (KPF-to-KAF) (G) and analyzed by confocal microscopy. Bar; 10 μm.

The KPF site in the unstructured EHD2 loop (Fig 1A and 1B) has been implicated in caveolar binding and stable EHD2 membrane assembly [22]. However, the impact of this PF motif on EHD2 homo-dimerization and interactions with binding partners has not been studied. Accordingly, we found that EHD2 KPF-to-APA or KPF-to-KAF mutants had no effect on dimerization or binding to Syndapin2 (Fig 5A–5D) by yeast two-hybrid binding assays. However, both KPF mutants reduced the level of EHD2 on the plasma membrane and increased the level of nuclear-localized EHD2 compared to wild-type EHD2 (Fig 5E–5G). For this reason, i.e., localization of the mutants primarily to the nucleus (as opposed to the plasma membrane), immunoprecipitation experiments did not detect binding between the mutants and Syndapin2. Overall, these studies suggest a complex function for the unstructured loop and its two PF motifs that may help supports the initial model [9,22] and revised model [23] proposed for dimer-pair PF-EH domain interactions and EHD2 oligomerization (see model for further discussion).

**Fig 6:**
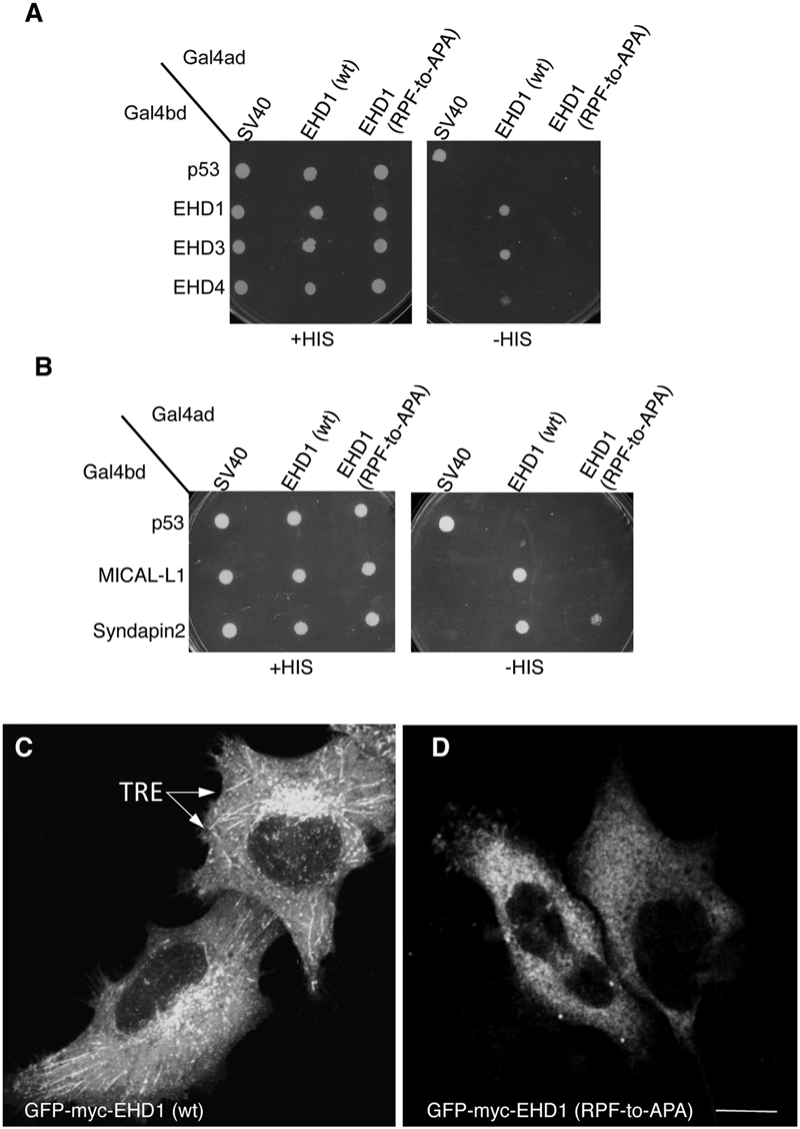
A single EHD1 PF motif (RPF) controls its homo- and hetero-dimerization, binding to interaction partners, and localization to Tubular Recycling Endosomes (TRE). (A) *S*. *cerevisae* yeast were co-transformed with Gal4bd fusion constructs: Gal4bd-p53 (control), -Syndapin-2 and -MICAL-L1 along with Gal4ad-SV40 (control), -EHD1 (wt.) and -EHD1 (RPF-to-APA). (B) As in (A), yeast were co-transformed with Gal4bd-p53 (control), -EHD1 (wt), -EHD3 (wt), -EHD4 (wt), along with Gal4ad-SV40 (control), -EHD1 (wt) and EHD1 (RPF-to-APA). Co-transformants from A-B were plated on non-selective (+HIS) and selective (-HIS) agar plates. (C–D) HeLa cells were grown on coverslips, transfected with GFP-myc-EHD1 (wt) (C), GFP-myc-EHD1 (RPF-to-APA) (D, arrows point to TRE). Bar; 10 μm.

Given that EHD1 contains a single PF motif, namely the RPF motif that aligns with the EHD2 KPF motif (see Fig 1B), we tested the role of this motif on EHD1 dimerization and partner binding. The EHD1 RPF-to-APA mutant displayed abrogated homo- and hetero-dimerization (Fig 6A), loss of binding to MICAL-L1, and decreased binding to Syndapin2 (Fig 6B). Moreover, the typical subcellular localization of EHD1 to an array of tubular and vesicular recycling endosomes (Fig 6C) became mostly cytoplasmic for EHD1 RPF-to-APA mutant (Fig 6D). These data suggest that the single RPF motif in EHD1 carries out the functions of the dual KPF and NPF motifs found in EHD2.

**Fig 7:**
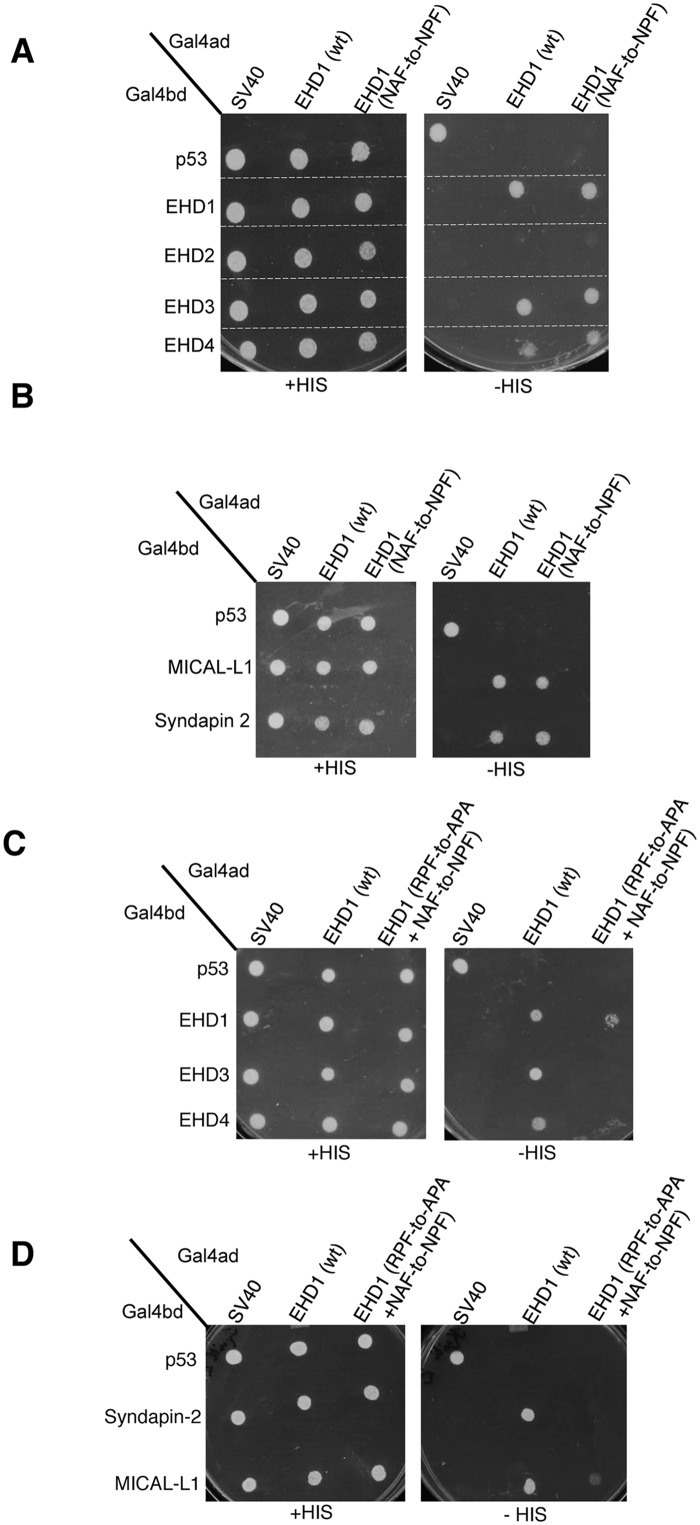
The NAF motif of EHD1 is dispensable for homo- or hetero-oligomerization, and for its association with binding partners. (A) *S*. *cerevisae* yeast were co-transformed with the following Gal4bd fusion constructs: Gal4bd-p53 (control), -MICAL-L1, and -Syndapin-2 along with Gal4ad-SV40 (control), -EHD1 (wt.) and -EHD1 (NAF to NPF). (B) As in (A), yeast were co-transformed with Gal4bd-p53 (control), -EHD1 (wt), -EHD2 (wt), -EHD3 (wt) and -EHD4 (wt), along with Gal4ad-SV40 (control), -EHD1 (wt) and -EHD1 (NAF-to-NPF). (C) As in (A), yeast were co-transformed with Gal4bd-p53 (control), -EHD1 (wt), -EHD3 (wt), and -EHD4 (wt), along with Gal4ad-SV40 (control), -EHD1 (wt) and -EHD1 (NAF-to-NPF). (D) Yeast were co-transformed with Gal4bd-p53 (control), -Syndapin-2, and -MICAL-L1, along with Gal4ad-SV40 (control), EHD1 (wt) and EHD1 (NAF-to-NPF). Co-transformants in A-D were plated on non-selective (+HIS) and selective (-HIS) agar plates.

Since EHD1 has a “non-functional” NAF motif aligned with the NPF motif of EHD2 (depicted in Fig 1B), we asked whether the EHD1 NAF motif could assume functionality if its lone existing PF motif (RPF) was impaired. As a control, we first engineered EHD1 NAF-to-NPF mutants and showed that as expected, homo- and hetero-dimerization along with MICAL-L1 and Syndapin2 binding remained stable (Fig 7A and 7B). Next, we engineered double mutants where the EHD1 RPF motif was disrupted (RPF-to-APA) and the NAF motif was ‘corrected’ to NPF. Compared to the EHD1 RPF-to-APA single mutants, which lost all of their dimerization and protein interactions, the additional NAF-to-NPF mutation partially rescued homo-dimerization (Fig 7C) and binding to MICAL-L1 (Fig 7D). These results hint that gene duplication and subtle alterations may have allowed the EHD proteins to fine-tune their interactions and subcellular functions.

**Fig 8:**
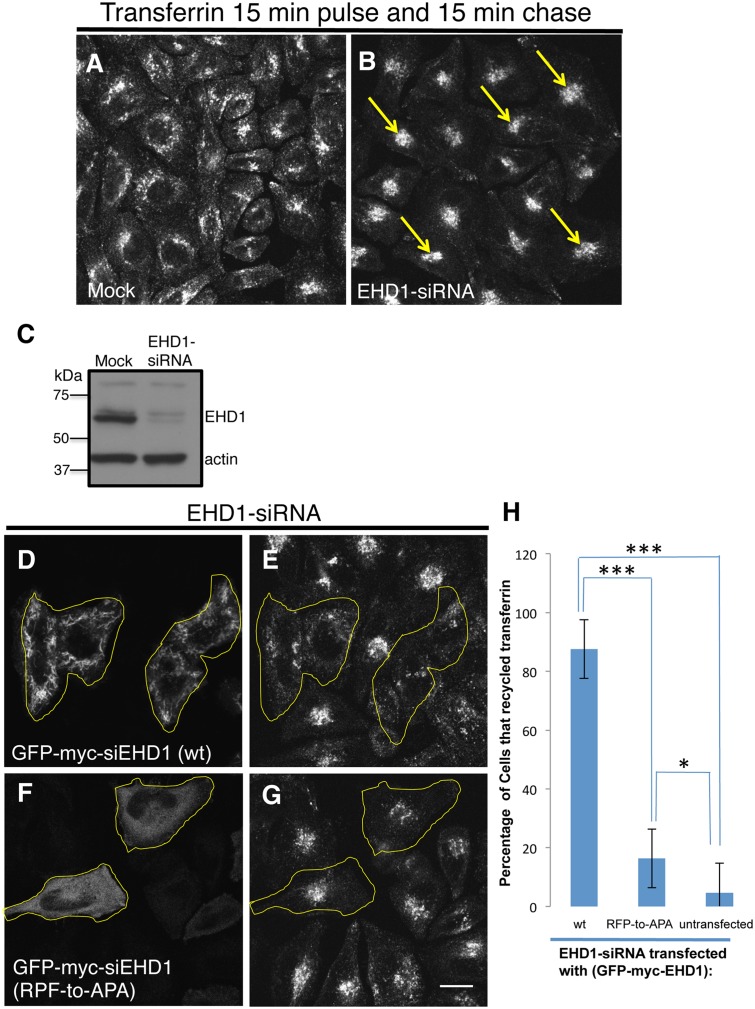
The EHD1 RPF motif is essential for receptor recycling. (A–B) HeLa cells grown on coverslips were mock-treated (A) or treated with EHD1-siRNA (B–F). For A–B, After a 30 min. serum-starvation, cells were allowed to uptake Tf-568 for 15 min., and then chased in complete media for 15 min. to permit Tf-568 recycling to the PM. (C) Immunoblot analysis of Mock- or EHD1-siRNA-treated cells depicting levels of actin (control) or EHD1. (D–G) During the EHD1-siRNA treatment, cells were transfected with the siRNA-resistant EHD1 constructs: siRNA-res-GFP-myc-EHD1 (wt) in (D), and siRNA-res-GFP-myc-EHD1 (RPF-to-APA) in (F). Following serum-starvation, Tf-568 was internalized for 15 min., and chased in complete media for 15 min. Note the typical concentration of Tf in the ERC in untransfected cells treated with EHD1-siRNA. (H) Quantitative analysis of 100 cells from 3 independent experiments as in (E and G). Cells that recycled Tf-568 (“empty of Tf”) were scored and calculated as % of total cell number, and portrayed with standard error bars. Stars represent significance of *p* < 0.01 (one star) or *p* < 0.05 (three stars) for one-way ANOVA tests. Bar; 10 μm.

To functionally assess the role of the EHD1 RPF motif, we used an siRNA approach coupled with rescue by transient transfection. As demonstrated, more than 90% of EHD1 was depleted upon treatment with EHD1-siRNA (Fig 8C). Compared to control cells, the depletion of EHD1 typically causes an accumulation of internalized transferrin and its receptor in the endocytic recycling compartment (ERC) when cells are then “chased” to allow the receptor and labeled ligand to recycle and exit the cell (compare Fig 8B with control Fig 8A). This accumulation is ‘rescued’ in EHD1-depleted cells in which a siRNA-resistant form of the wild-type EHD1 (siEHD1) has been transfected (Fig 8D and 8E; see yellow borders for transfected cells). On the other hand, when a cytoplasmic-localized EHD1 RPF-to-APA mutant was reintroduced into EHD1-depleted cells, the transferrin and its receptor remained accumulated in the perinuclear region of the cell (Fig 8F and 8G; see yellow borders for transfected cells). These data were quantified (from 3 experiments) and showed that whereas wild-type EHD1 rescued the recycling defect in >85% of the EHD1-depleted cells, the RPF-to-APA mutant displayed less than 15% rescue (Fig 8H).

Despite being the only EHD protein whose crystal structure has been solved [9], and despite important structural characterizations of its interaction with membranes [23] and its recruitment by PI(4,5)P2 to the plasma membrane [20], many of the mechanistic aspects by which EHD2 functions have remained obscure. In this study we have discovered intriguing differences in the function of the proline and phenylalanine residues of the EHD2 NPF motif that is found within the unstructured loop of the G-domain (Fig 1A). Our data suggest that the *phenylalanine* is crucial for localization to the plasma membrane, and mutation of this residue leads to its accumulation in the nucleus. On the other hand, mutation of the *proline* residue of this same NPF motif leads to loss of homo-oligomerization and presumed loss of oligomerization, as well as loss of protein partner binding without impacting localization to the plasma membrane.

**Fig 9:**
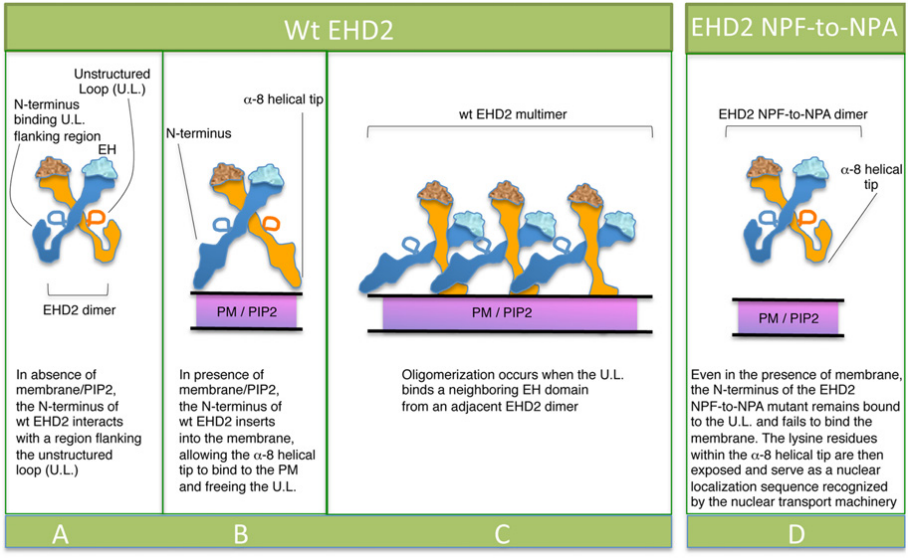
Model for the role of the EHD2 unstructured KPFRKLNPF motif in subcellular localization. (A) In the absence of PIP2-containing membrane, the N-termini interact with regions flanking the unstructured loops (U.L.) of EHD2 dimer pairs. (B) PIP2-binding by the N-terminus facilitates a-8 helical binding to the membrane and frees the U.L. (C) the U.L. NPF motif is now capable of interacting with an EH domain of a neighboring EHD2 dimer pair, inducing oligomerization. (D) For EHD2 NPF-to-NPA mutants, we hypothesize that the N-terminus may not insert into membranes, thus preventing the U.L. from oligomerizing and maintaining the lysine residues on α-8 helix free to serve as a nuclear localization sequence.

How may these data be interpreted given the elegant structural studies that propose PF-EH domain interactions for EHD2 oligomerization? Shah and colleagues recently proposed that upon membrane sensing, the EHD2 N-terminus moves away from the unstructured loop (U.L.) in the G-domain (illustrated in Fig 9A–9B), thus freeing the KPFRKLNPF (U.L.) for interactions with EH domains of nearby EHD2 dimer pairs, promoting oligomerization and caveolar localization (Fig 9C) [23]. Based on this model, we hypothesize that the N-terminal of the EHD2-NPF-to-NPA mutant (Fig 9D) may not completely detach from the G-domain regions flanking the U.L. and thus fail to insert properly into the plasma membrane. We rationalize that under such conditions where membrane binding is partially impaired, the lysine residues in the EHD2 helical domain, which act both as membrane contact sites [9] and a bipartite nuclear localization sequence [27], are now free to act in the latter capacity and promote EHD2 transport to the nucleus. Indeed, EHD2 has been described as a transcriptional repressor [27].

On the other hand, we predict that with the EHD2 NPF-to-NAF mutant, the EHD2 N-terminus is nonetheless freed from the G-domain to interact with the plasma membrane. However, despite its correct localization, the NAF motif prevents EH binding and thus dimerization and oligomerization do not occur.

Overall, our study provides support and additional new details that further clarify the complex structural model of EHD2 localization, membrane binding, protein binding, dimerization, oligomerization, and function.

## Supporting Information

S1 Fig EHD dimerization requires an intact α-helical region, but not the presence of EH domains. (A) Schematic diagram of EHD2 domain organization. (B–C) Yeast were co-transformed with Gal4bd fusion constructs: Gal4bd-p53 (control), EHD2 (wt), EHD2 (ΔEH) along with Gal4ad-SV40 (control), EHD2 (wt), EHD2 (ΔEH), EHD1 (wt), EHD1 (ΔEH), and EHD1. All co-transformants were plated on non-selective (+HIS) and selective (-HIS) media.
(TIF)

S2 Fig Co-immunoprecipitation of EHD2 and mutant EHD2 proteins with Syndapin2. HeLa cells were transfected with either GFP-Myc-EHD2, or GFP-Myc-EHD2 mutants (NPF-to-NAF, NPF-to-APA, KPF-to-KAF, KPF-to-APA), lysed 24 h later and subjected to immunoprecipitation with antibodies against Syndapin2. Separated proteins were transferred onto nitrocellulose and then immunoblotted with anti-EHD2 antibodies. Input contains 5% of the total lysate immunoprecipitated.
(PPTX)

S3 Fig Wild-type and EHD2 NPF-to-NPY homo-dimerize and interact with Syndapin2, whereas EHD2 NPF-to-NFP does not. *S*. *cerevisae* yeast were co-transformed with the following Gal4bd fusion constructs: Gal4bd-p53 (control), -EHD2 (wt), -MICAL-L1, and -Syndapin-2 along with Gal4ad-SV40 (control), -EHD2 (wt), -EHD2 NPY, and EHD2 NFP. Co-transformants in were plated on non-selective (+HIS) and selective (-HIS) agar plates.
(PPTX)

S1 Table Comparison of wild-type EHD2 and mutants in homo-dimerization, Syndapin2-binding and sub-cellular localization. (PPTX)
