# Supplementary figures and images for: Role of the EHD2 Unstructured Loop in Dimerization, Protein Binding and Subcellular Localization
Source: PLoS One. 2015 Apr 15;10(4):e0123710. doi: 10.1371/journal.pone.0123710 (PMC4398442; doi:10.1371/journal.pone.0123710)

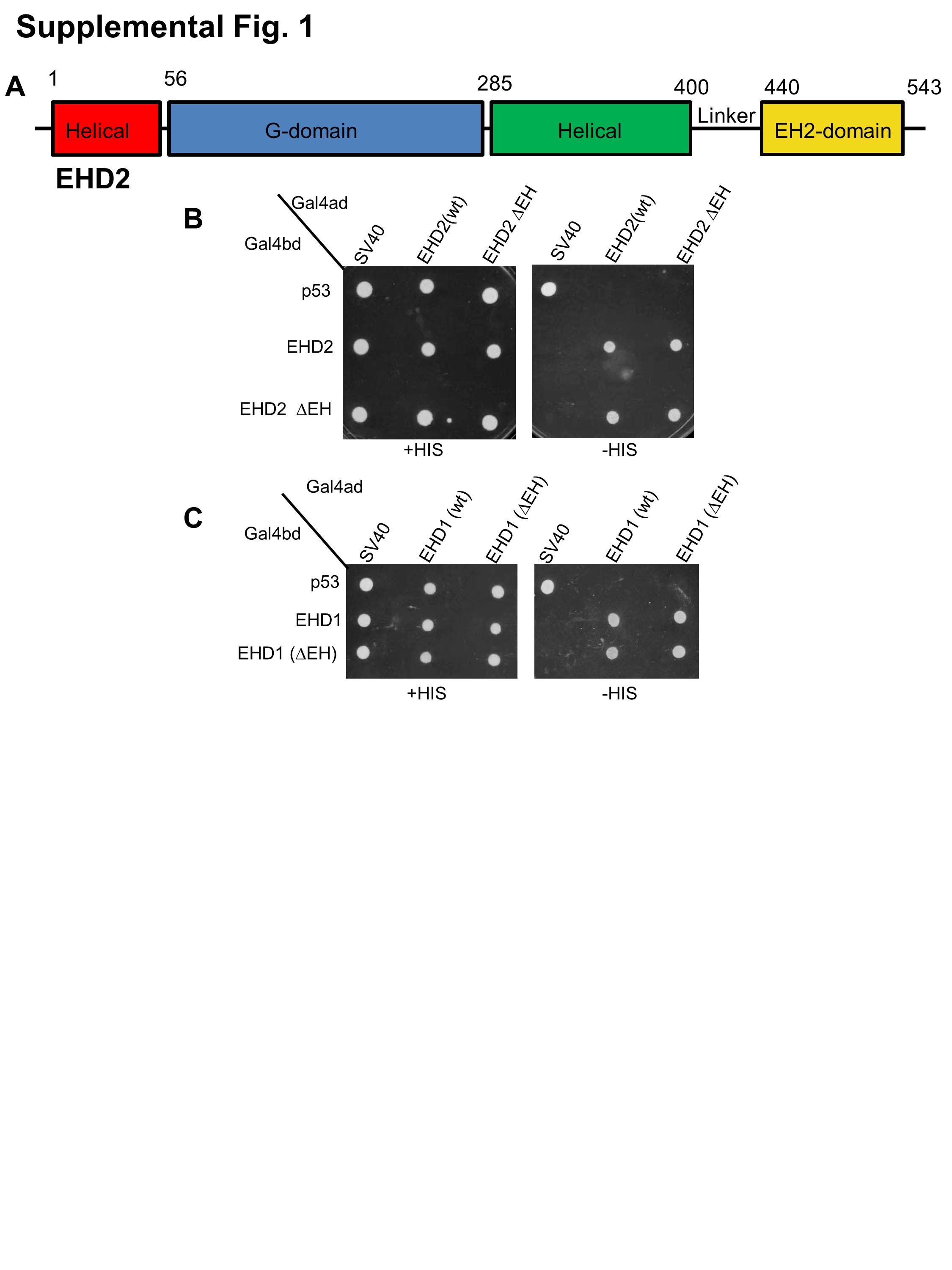

Supplement: S1 Fig — (A) Schematic diagram of EHD2 domain organization. (B–C) Yeast were co-transformed with Gal4bd fusion constructs: Gal4bd-p53 (control), EHD2 (wt), EHD2 (ΔEH) along with Gal4ad-SV40 (control), EHD2 (wt), EHD2 (ΔEH), EHD1 (wt), EHD1 (ΔEH), and EHD1. All co-transformants were plated on non-selective (+HIS) and selective (-HIS) media. (TIF) [file pone.0123710.s001.tif]
