# Supplementary material for: Role of the EHD2 Unstructured Loop in Dimerization, Protein Binding and Subcellular Localization
Source: PLoS One. 2015 Apr 15;10(4):e0123710. doi: 10.1371/journal.pone.0123710 (PMC4398442; doi:10.1371/journal.pone.0123710)
Supplement: S2 Fig — HeLa cells were transfected with either GFP-Myc-EHD2, or GFP-Myc-EHD2 mutants (NPF-to-NAF, NPF-to-APA, KPF-to-KAF, KPF-to-APA), lysed 24 h later and subjected to immunoprecipitation with antibodies against Syndapin2. Separated proteins were transferred onto nitrocellulose and then immunoblotted with anti-EHD2 antibodies. Input contains 5% of the total lysate immunoprecipitated. (PPTX) [file pone.0123710.s002.pptx]

## Slide 1
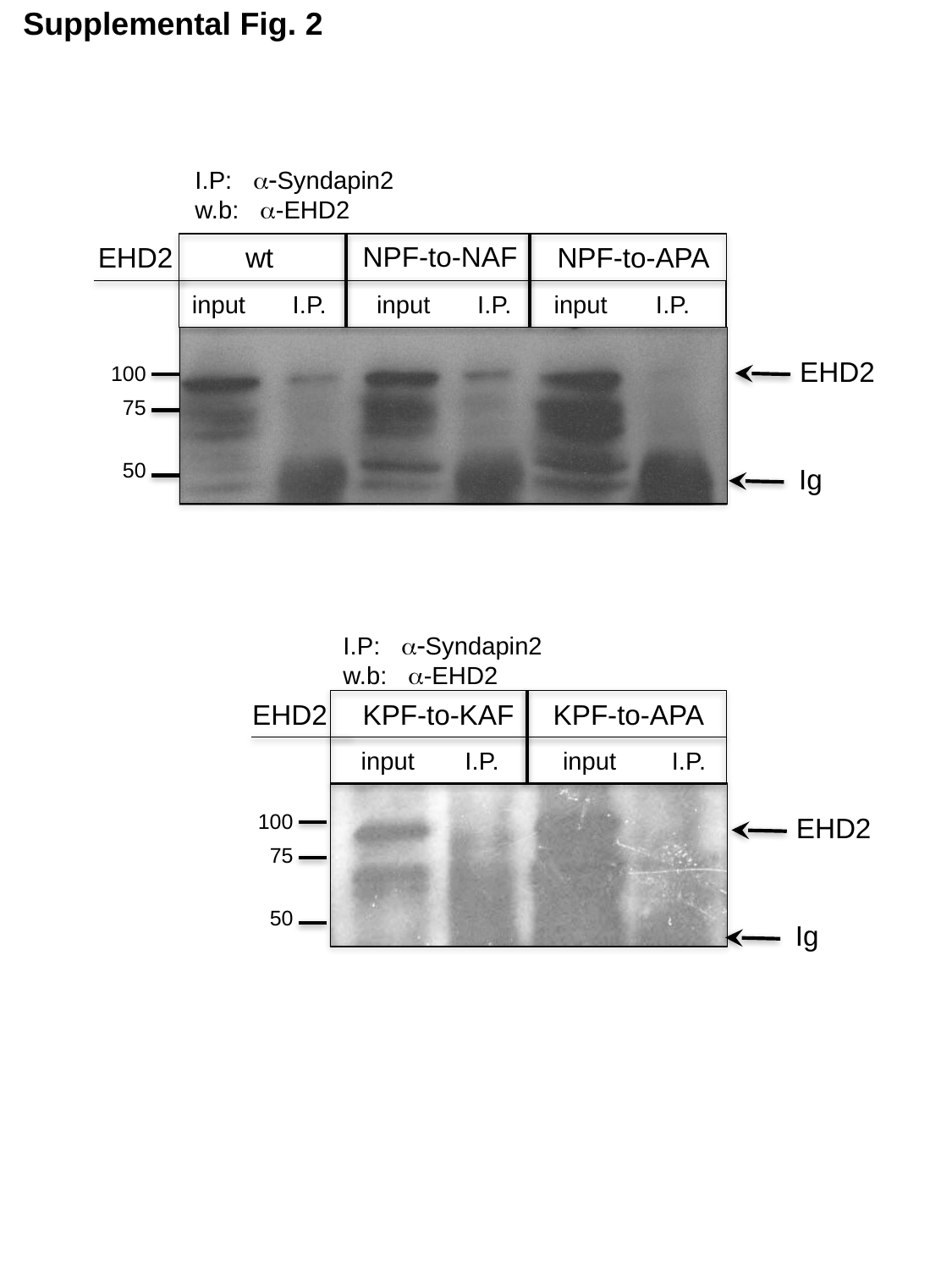

Supplemental Fig. 2
I.P: a-Syndapin2
w.b: a-EHD2
NPF-to-NAF
EHD2
wt
NPF-to-APA
input
I.P.
input
I.P.
input
I.P.
EHD2
100
75
50
Ig
I.P: a-Syndapin2
w.b: a-EHD2
EHD2
KPF-to-KAF
KPF-to-APA
input
I.P.
input
I.P.
100
75
50
EHD2
Ig
