# Supplementary material for: Role of the EHD2 Unstructured Loop in Dimerization, Protein Binding and Subcellular Localization
Source: PLoS One. 2015 Apr 15;10(4):e0123710. doi: 10.1371/journal.pone.0123710 (PMC4398442; doi:10.1371/journal.pone.0123710)
Supplement: S3 Fig — S. cerevisae yeast were co-transformed with the following Gal4bd fusion constructs: Gal4bd-p53 (control), -EHD2 (wt), -MICAL-L1, and -Syndapin-2 along with Gal4ad-SV40 (control), -EHD2 (wt), -EHD2 NPY, and EHD2 NFP. Co-transformants in were plated on non-selective (+HIS) and selective (-HIS) agar plates. (PPTX) [file pone.0123710.s003.pptx]

## Slide 1
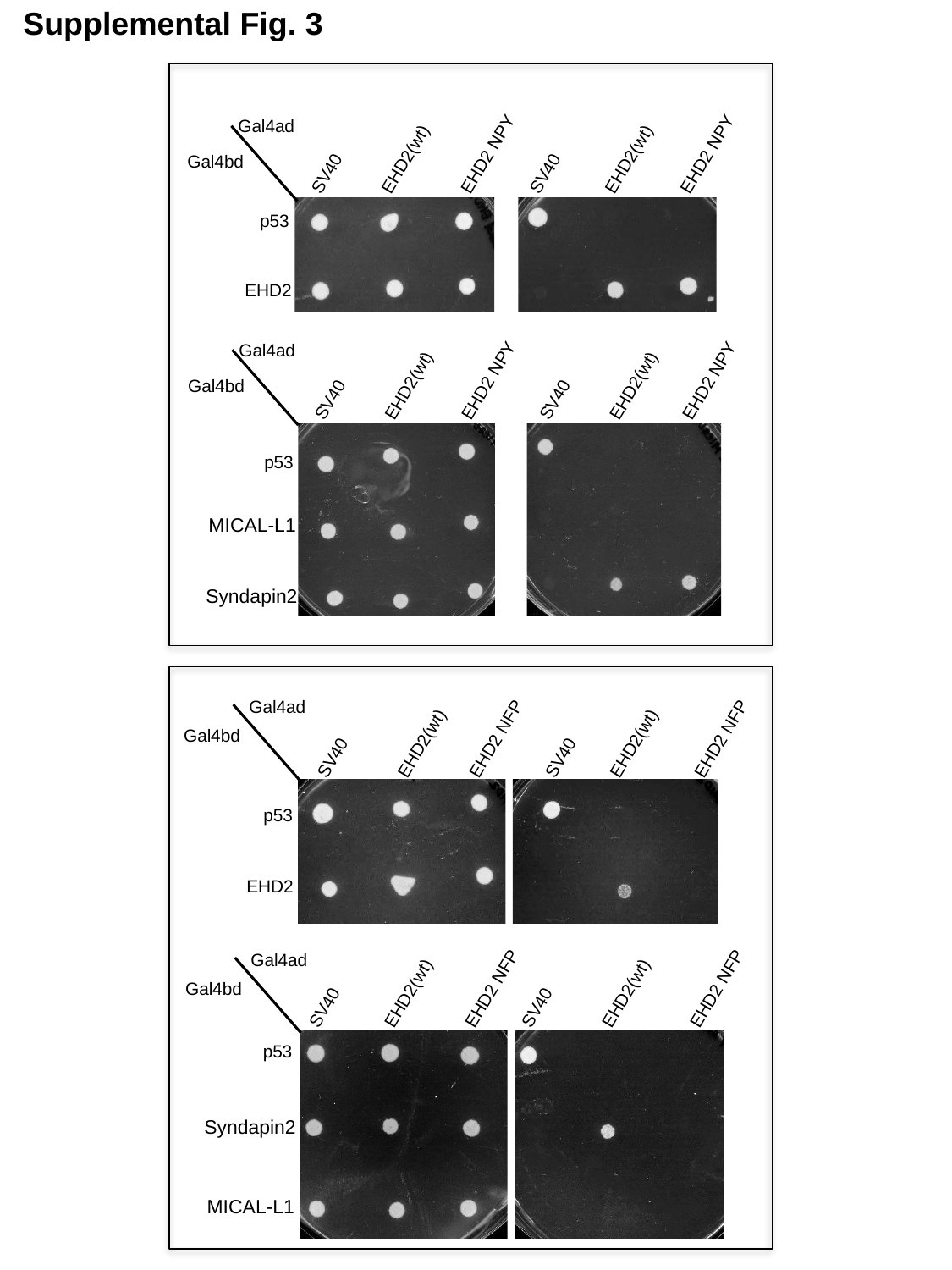

Supplemental Fig. 3
Gal4ad
Gal4bd
EHD2(wt)
EHD2 NPY
EHD2(wt)
EHD2 NPY
SV40
SV40
 p53
 EHD2
Gal4ad
Gal4bd
EHD2(wt)
EHD2 NPY
SV40
 p53
MICAL-L1
 Syndapin2
EHD2(wt)
EHD2 NPY
SV40
Gal4ad
Gal4bd
EHD2(wt)
EHD2 NFP
SV40
 p53
 EHD2
EHD2(wt)
EHD2 NFP
SV40
Gal4ad
Gal4bd
EHD2(wt)
EHD2 NFP
SV40
 p53
 Syndapin2
MICAL-L1
EHD2(wt)
EHD2 NFP
SV40
