# Supplementary material for: Role of the EHD2 Unstructured Loop in Dimerization, Protein Binding and Subcellular Localization
Source: PLoS One. 2015 Apr 15;10(4):e0123710. doi: 10.1371/journal.pone.0123710 (PMC4398442; doi:10.1371/journal.pone.0123710)
Supplement: S1 Table — (PPTX) [file pone.0123710.s004.pptx]

## Slide 1
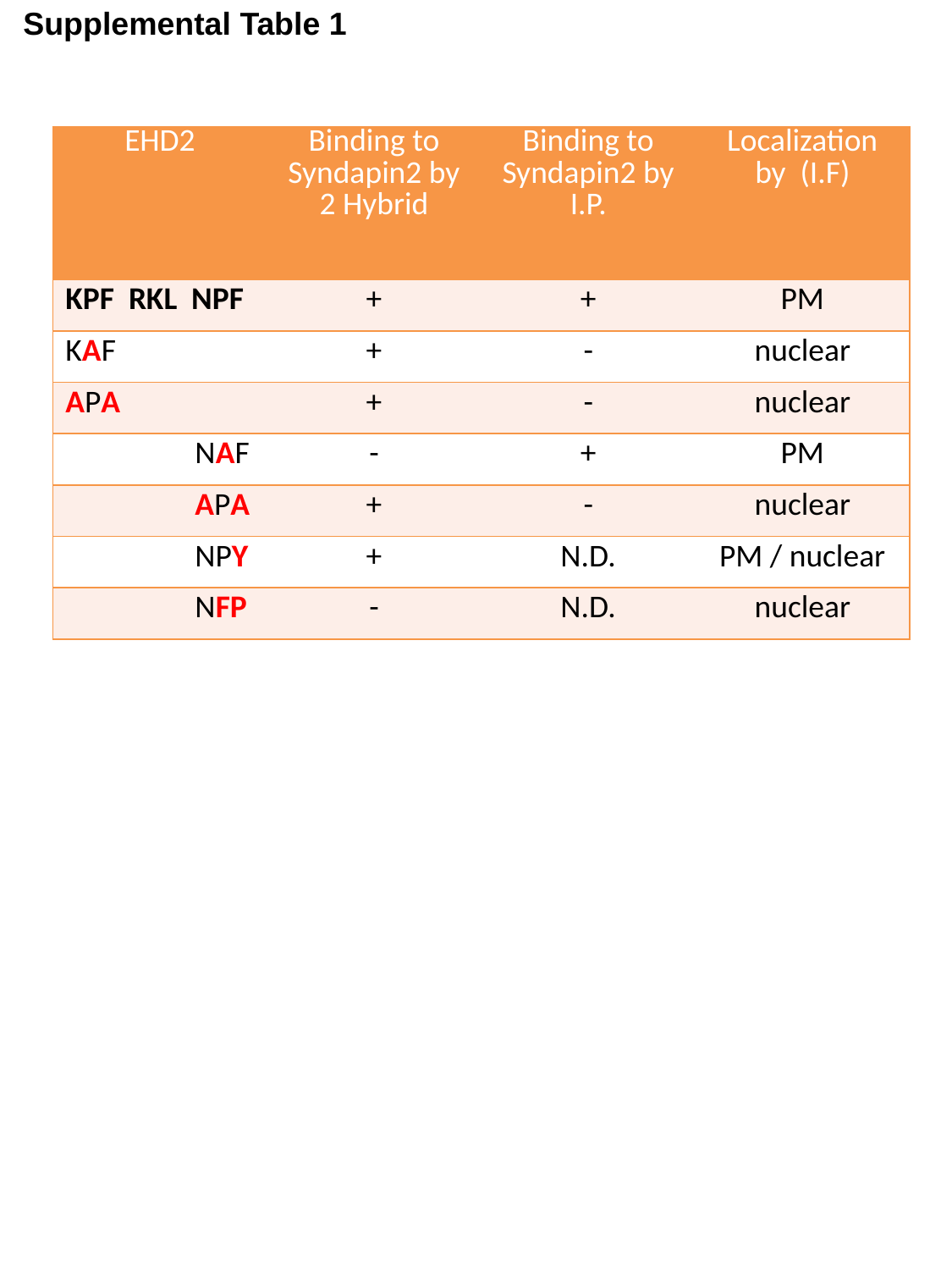

Supplemental Table 1
| EHD2 | Binding to Syndapin2 by 2 Hybrid | Binding to Syndapin2 by I.P. | Localization by (I.F) |
| --- | --- | --- | --- |
| KPF RKL NPF | + | + | PM |
| KAF | + | - | nuclear |
| APA | + | - | nuclear |
| NAF | - | + | PM |
| APA | + | - | nuclear |
| NPY | + | N.D. | PM / nuclear |
| NFP | - | N.D. | nuclear |
